# Acquired von Willebrand Syndrome in IgM Monoclonal Gammopathy as the Presentation of Lymphoplasmacytic Lymphoma

**DOI:** 10.1155/2017/9862620

**Published:** 2017-05-29

**Authors:** Zachary Wolfe, Bradley Lash

**Affiliations:** ^1^Department of Internal Medicine, Guthrie Clinic/Robert Packer Hospital, Sayre, PA, USA; ^2^Department of Hematology and Oncology, Guthrie Clinic/Robert Packer Hospital, Sayre, PA, USA

## Abstract

Acquired von Willebrand syndrome (AVWS) is an increasingly recognized entity with numerous potential underlying etiologies. Most commonly implicated are lymphoproliferative, myeloproliferative, cardiovascular, and autoimmune disorders. Unlike inherited von Willebrand disease (vWD), AVWS tends to present at an older age and without a family history of vWD. Treatment is directed at the underlying etiology if one is uncovered, as well as treatment and prevention of bleeding. Here, we present a rare case of AVWS secondary to Waldenström macroglobulinemia which went unrecognized for several years but resolved promptly with treatment. The potential mechanisms of AVWS secondary to monoclonal gammopathies are discussed as well as strategies to treat and prevent bleeding in these patients.

## 1. Introduction

Recognition of acquired von Willebrand syndrome (AVWS) and its many causes, including malignancy, is important and a high level of suspicion for AVWS must be maintained in the workup of bleeding disorders. When AVWS is suspected, there are numerous underlying etiologies that must be considered including both hematologic and nonhematologic neoplasms, cardiovascular disorders, autoimmune diseases, and drugs among other less frequent causes such as hypothyroidism and uremia [1, 2]. Uncovering the most likely underlying etiology allows for the most effective management [2]. We report the case of a woman with an uncommon malignant etiology of AVWS that remained undiagnosed for ten years. The AVWS resolved with treatment of the underlying malignancy.

## 2. Case Description

A 61-year-old female with a past medical history of presumed type 2 von Willebrand disease (vWD) diagnosed at age of 51 and IgM-kappa monoclonal gammopathy of undetermined significance (MGUS) diagnosed one year later presented to the hematology clinic with increasing frequency and severity of epistaxis. Numerous prolonged episodes of epistaxis had been successfully treated with VWF/factor VIII replacement. Her original diagnosis of vWD was made after referral to hematology because of bleeding for eight hours after a dental extraction. She also had prolonged bleeding after removal of a basal cell carcinoma from her right arm at the age of 46. Prior to the age of 46, she had not experienced any prolonged bleeding with dental extractions and she had never undergone surgery.

**Figure 1:**
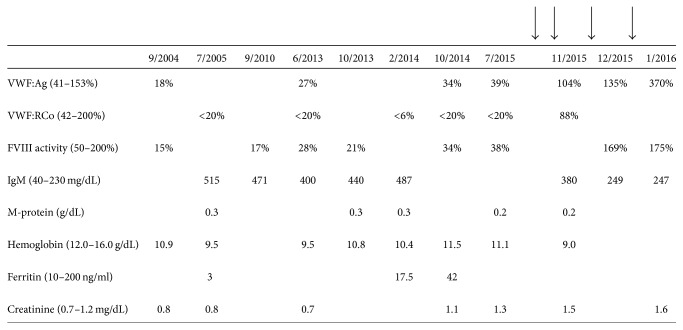
Laboratory response of AVWS after treatment of lymphoplasmacytic lymphoma with carfilzomib, rituximab, and dexamethasone. Arrows indicate cycles of treatment. Treatment resulted in normalization of VWF antigen level (VWF:Ag), VWF activity (VWF:RCo), and factor VIII activity assay (FVIII activity). Normal values are shown in parentheses.

Review of her baseline laboratory studies revealed that her von Willebrand factor antigen (VWF:Ag) was 18% (normal: 41–153%), ristocetin cofactor activity (VWF:RCo) was <20% (normal: 42–200%), and factor VIII activity was 34% (normal: 50–200%). Multimeric analysis showed absence of high molecular weight von Willebrand factor. SPEP revealed a narrow band in the gamma area consistent with monoclonal gammopathy and an M-protein of 0.3 g/dL which was confirmed to be IgM-kappa on immunofixation. Serial IgM concentrations are shown in Figure 1. Her CBC revealed mild normocytic anemia with a hemoglobin of 11.1 g/dL (normal: 12.0–16.0 g/dL) and an MCV of 90.8 fL (normal: 80.0–100.0 fL). In the past, she had iron deficiency anemia which had responded to oral iron replacement (see Figure 1 for serial ferritin and hemoglobin levels). Her platelet and leukocyte counts were normal with normal white blood cell differential counts on CBC. Serum creatinine is shown in Figure 1. She had no evidence of liver dysfunction based on normal INR, bilirubin, and albumin levels.

**Figure 2:**
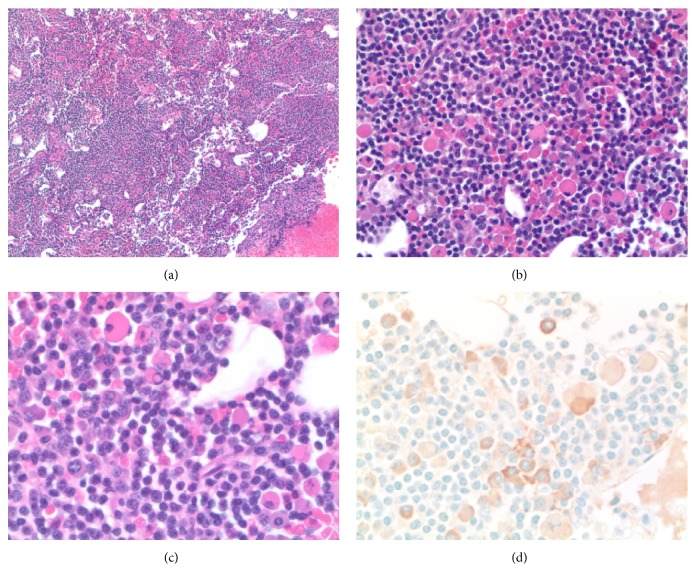
Lymphoplasmacytic lymphoma, H&E (a). Plasma cells with Dutcher bodies (b). Lymphocytes and plasma cells with Dutcher bodies and Russell bodies (c). Kappa light chain stain (d).

Notably, she had no family history of von Willebrand disease. Because of her lack of family history and her older age at diagnosis, we suspected that she had an acquired von Willebrand syndrome rather than inherited von Willebrand disease. Furthermore, we suspected that the underlying etiology of the AVWS was the monoclonal gammopathy. Although she had a normal bone marrow biopsy at the time the MGUS was diagnosed and her M-protein concentration had remained stable over time (Figure 1), her bleeding symptoms were becoming more frequent and there was a possibility of sampling error of the first bone marrow biopsy. Therefore, we performed a repeat bone marrow aspiration and biopsy to search for an underlying lymphoproliferative disorder which could guide our management of this patient. Bone marrow biopsy now showed 70% involvement by lymphoplasmacytic lymphoma (LPL) (Figure 2). A diagnosis of Waldenström macroglobulinemia was made.

Treatment was initiated with carfilzomib, rituximab, and dexamethasone (CaRD). We anticipated that treatment of the LPL and reduction of the monoclonal protein would also treat the von Willebrand syndrome, confirming that it was secondary to the monoclonal gammopathy and LPL. Figure 1 shows the timing of her treatment and the concomitant changes in VWF:Ag, VWF:RCo, and FVIII activity. After two cycles of CaRD, these levels returned to normal and she had no further clinically significant bleeding.

While her AVWS had resolved, she died four months later after developing a multifocal pneumonia secondary to invasive aspergillosis with CNS involvement. Despite treatment with voriconazole and caspofungin, she did not improve and she became obtunded and required intubation and mechanical ventilation. In keeping with her wishes, at the request of her family, she was placed on comfort care measures only at which time she died.

## 3. Discussion

Suspicion for AVWS should be high in patients who present with symptoms attributable to abnormal von Willebrand factor at an older age and without a family history of vWD. Once AVWS is suspected, one must search thoroughly for the underlying etiology so that reversible causes can be treated. In this case, AVWS had been presumed to be inherited vWD and the association with the IgM monoclonal gammopathy went unrecognized for several years. While only about 2–4% of AVWS cases are associated with LPL [3], 13% of patients with LPL had laboratory evidence for AVWS in one series [4].

Numerous etiologies of AVWS have been observed with several different reported mechanisms [1]. Interestingly, a single underlying disorder may have several potential mechanisms for causing AVWS [1, 2]. Except in the case of hypothyroidism [5], the described mechanisms do not appear to affect the production or release of von Willebrand factor but rather lead to increased clearance and/or decreased function [1, 2]. Increased shear stress leading to destruction of vWF, especially high molecular weight multimers, is implicated in AVWS secondary to cardiac causes such as aortic stenosis and ventricular-assist devices [6, 7]. This is also seen in AVWS secondary to certain paraproteinemias as described in more detail below. Autoantibodies can lead to increased clearance of vWF via the formation of immune complexes or decreased vWF function by interference with functional domains related to collagen or platelet glycoprotein binding [8, 9]. This mechanism is implicated in autoimmune etiologies such as systemic lupus erythematosus as well as in lymphoproliferative disorders [1, 5]. Myeloproliferative disorders, especially essential thrombocythemia and polycythemia vera, can cause AVWS via loss of high molecular weight vWF multimers [5].

Potential mechanisms for AVWS associated with MGUS include vWF-specific inhibitors [3, 4, 8, 10, 11], adsorption of vWF multimers onto malignant cells [3, 4, 10, 12], formation of immune complexes between vWF and nonspecific antibodies [10, 13], and increased degradation of vWF multimers [4, 14]. The mechanism may be different for IgG-MGUS versus IgM-MGUS based on different responses to treatment with IVIG [10, 13] and different patterns of vWF abnormalities, especially loss of high molecular weight multimers in IgM but not IgG monoclonal gammopathies [10]. In Waldenström macroglobulinemia, AVWS is associated with higher IgM levels, cryoglobulinemia, and higher serum viscosity [4]. High serum viscosity increases shear force which has been shown to cause destruction of large vWF multimers by changing their conformation in a way that enhances their susceptibility to cleavage by ADAMTS13 [4, 15, 16].

Management of AVWS focuses on treating the underlying etiology, treatment of bleeding, and prevention of bleeding in high risk situations (i.e., surgery). In AVWS associated with IgM-MGUS or IgG-MGUS, desmopressin (DDAVP) or vWF/factor VIII replacement can be used to both treat and prevent bleeding by transiently improving the bleeding time and increasing factor VIII and vWF levels [10]. Infusion of vWF/factor VIII concentrate has been shown to normalize bleeding time within 30 minutes of infusion, but by two hours the bleeding time starts to increase and reaches the preinfusion baseline by four hours [10]. IVIG provides more sustained improvement in bleeding time for AVWS secondary to IgG-MGUS but not IgM-MGUS [10, 13, 14]. IVIG given 1 g/kg/day for two days can cause normalization of the bleeding time and factor VIII and vWF levels beginning one day after infusion [10]. Because of the time it takes for bleeding time to normalize after IVIG, vWF/factor VIII replacement or DDAVP may still be necessary if the patient is bleeding [2]. Normal levels are sustained for nearly 18 days after IVIG and there is return to baseline by day 21 [10, 11]. IVIG may be particularly helpful in cases where direct inhibitors of vWF are found [11]. In Waldenström macroglobulinemia with a high serum viscosity, plasma exchange is another treatment option. However, its usefulness is limited because multiple plasma exchanges are required and normalization of VWF:RCo can take up to 10 days [4].

The cornerstone of treatment for AVWS is to treat the underlying disorder. However, in the case of AVWS secondary to a monoclonal gammopathy, when the patient does not meet criteria for multiple myeloma or LPL, treatment remains focused on prevention and treatment of bleeding. The monoclonal gammopathy itself is usually only treated once criteria for multiple myeloma or LPL are met. One case report of AVWS associated with IgG-MGUS showed no response to treatment with rituximab [17]. However, one case report of AVWS secondary to IgG-MGUS reported successful treatment of the AVWS with normalization of vWF levels and activity using bortezomib and dexamethasone [18]. However, this was a single case and whether or not one should treat the presumed plasma cell dyscrasia underlying a monoclonal gammopathy of undetermined significance causing AVWS remains a topic for future research. Our case adds to the evidence that it should be considered, although it is important to note that the CaRD treatment may have predisposed our patient to the development of invasive aspergillosis. This highlights the need to consider the risks of treatment and weigh them against the benefits.

In summary, distinguishing between inherited von Willebrand disease and acquired von Willebrand syndrome is an important diagnostic step in the patient presenting with von Willebrand factor abnormalities. This case teaches that AVWS may go unrecognized and delay effective treatment, that treating underlying lymphoproliferative disorders can effectively cure AVWS, and that one must weigh the risks and benefits carefully before proceeding with such treatment. Prevention and treatment of bleeding in AVWS secondary to an IgM or IgG monoclonal gammopathy can be accomplished with DDAVP and/or vWF/factor VIII replacement products. IVIG is an option that appears to be effective only in cases secondary to IgG monoclonal proteins. As our case demonstrates, definitive treatment of the AVWS may be accomplished by treating the plasma cell myeloma or LPL underlying the monoclonal gammopathy, if criteria for one of these conditions are met. Future research should address whether treating the plasma cell dyscrasia even when these criteria are not met can improve outcomes in the patient with AVWS secondary to a monoclonal protein. This should especially be considered in the patient who does not respond well to the conventional management of bleeding as described above or in the patient who requires such frequent treatment that it becomes more practical to treat the underlying disease, as demonstrated by our case. The best treatment regimen in such a situation is unknown but should carefully account for potential toxicities such that treatment toxicity does not outweigh the benefit of potentially curing the AVWS. If bleeding symptoms are severe, more intense regimens may be warranted to increase the likelihood of resolving the AVWS despite their increased toxicity. More experience with these situations is needed to help clarify what regimens may be used.
